# Effects of an mHealth voice message service (mMitra) on maternal health knowledge and practices of low-income women in India: findings from a pseudo-randomized controlled trial

**DOI:** 10.1186/s12889-020-08965-2

**Published:** 2020-06-01

**Authors:** Nirmala Murthy, Subhashini Chandrasekharan, Muthu Perumal Prakash, Aakash Ganju, Joanne Peter, Nadi Kaonga, Patricia Mechael

**Affiliations:** 1 Foundation for Research in Health Systems, #G-1, Brigade Business Suites, 10th Main, Jayanagar 2nd Block, Bengaluru, 560011 India; 2 HealthEnabled, Unit D11 Westlake Square, Westlake Drive, Westlake, Cape Town, 7945 South Africa; 3 National Institutes of Health, 6011 Executive Blvd, Rockville, MD USA; 4 Foundation for Research in Health Systems, G2, 5/26 Pillayar Kovil Street, Medavakkam, Chennai, 600100 India; 5 Saat Health, 1103, Glencroft, Hiranandani Gardens, Powai, Mumbai, 400076 India; 6 Johnson & Johnson, 241 Main Road, Retreat, Cape Town, 7945 South Africa; 7 Tufts University School of Medicine, 145 Harrison Ave, Boston, MA 02111 USA

**Keywords:** mHealth, Voice messaging, Low- and middle-income countries, Maternal care practices, Maternal care knowledge

## Abstract

**Background:**

Mobile Health (mHealth) is becoming an important tool to improve health outcomes in maternal, newborn and child health (MNCH). Studies of mHealth interventions, have demonstrated their effectiveness in improving uptake of recommended maternal services such as antenatal visits. However, evidence of impact on maternal health outcomes is still limited.

**Methods:**

A pseudo-randomized controlled trial (single blind) was conducted to assess the impact of a voice-message based maternal intervention on maternal health knowledge, attitudes, practices and outcomes over time: Pregnancy (baseline/Time 1); Post-partum (Time 2) and when the infant turned one year old (Time 3). Women assigned to the mMitra intervention arm received gestational age- and stage-based educational voice messages via mobile phone in Hindi and Marathi, while those assigned to the control group did not. Both groups received standard care.

**Results:**

Two thousand sixteen women were enrolled. Interviews were conducted with 1516 women in the intervention group and 500 women in the control group at baseline and post-partum. The intervention group performed significantly better than controls on four maternal health practice indicators: receiving the tetanus toxoid injection (OR: 1.6, 95% Confidence Interval (CI): 1.05–2.4, *p* = 0.028), consulting a doctor if spotting or bleeding (OR: 1.72, 95%CI: 1.07–2.75, *p* = 0.025), saving money for delivery expenses (OR: 1.79, 95%CI: 1.38–2.33, *p* = 0.0001), and delivering in hospital (OR: 2.5, 95%CI: 1.49–4.35, *p* = 0.001). The control group performed significantly better than the intervention group on two practice indicators: resting regularly during pregnancy (OR: 0.7, 95%CI: 0.54–0.88, *p* = 0.002) and having at-home deliveries attended by a skilled birth attendant (OR: 0.46, 95%CI: 0.23–0.91, *p* = 0.027). Both groups’ knowledge improved from Time 1 to Time 2. Only one knowledge indicator, on seeking medical care during pregnancy, was statistically increased in the intervention group compared to controls. Anemia status at or near the time of delivery was unable to be assessed due to missing data from maternal health cards.

**Conclusions:**

This study provides evidence that in low-resource settings, mobile voice messages providing tailored and timed information about pregnancy can positively impact maternal health care practices proven to improve maternal health outcomes. Additional research is needed to assess whether voice messaging can motivate behavior change better than text messaging, particularly in low literacy settings.

**Trial registration:**

The mMitra impact evaluation is registered with ISRCTN under Registration # 88968111, assigned on 6 September 2018 (See **https://www.isrctn.com/ISRCTN88968111**).

## Background

Urban slums are home to some of the poorest and most under-served populations. Due to living conditions and limited access to care, women and children in these settings often die from preventable causes. This is especially notable in India, which has some of the world’s largest slums with high burdens of disease and increasing mobile phone penetration rates [1]. Simple measures, such as vaccinations, antibiotics, improved nutrition and/or supplementation are proven interventions that can improve health outcomes. However, generating demand and promoting healthy behaviors are challenging.

Mobile health (mHealth), or the use of mobile technology for health, is becoming an important mechanism to improve maternal, neonatal and child health (MNCH) globally. Through targeted client communication, mobile phones enable pregnant women to access and/or receive information – whether via hotlines, direct messaging or smartphone applications – that can potentially lead to improved uptake of MNCH services and, subsequently, health outcomes. Additionally, mobile phones have also helped reach individuals who do not normally engage with the health system. Systematic reviews assessing the effectiveness of mHealth interventions on maternal and child health in low- and middle-income countries (LMICs) have shown that mHealth can improve maternal health service uptake, retention in care, adherence to treatment, and use of facility-based services such as antenatal care (ANC) visits, but the studies recommend that more research is needed to assess the impact of mHealth on clinical outcomes [2–5].

Accordingly, a pseudo-randomized controlled trial was conducted to test the hypothesis that a mobile phone-based voice messaging service in India, called mMitra, would lead to improved antenatal care (ANC) practices and maternal self-care knowledge and consequently better maternal health outcomes – specifically lower levels of anemia in women- a priority maternal health outcome in India [6, 7]. Despite significant progress between 1990 and 2017, India’s overall maternal mortality at the time of the study was till high at 145 per 100,000 [8].

## Methods

From January 2015 to December 2017, a pseudo-randomized controlled trial was conducted to evaluate the impact of mMitra. mMitra is the India country program affiliated with the Mobile Alliance for Maternal Action (MAMA), a 4-year global initiative that focused on improving the health and wellbeing of pregnant women and their newborns and infants through mobile phone delivered age- and stage-based tailored voice or text messages [7]. Through support from MAMA, the non-profit Advancing Reduction in Mortality and Morbidity of Mothers, Children and Neonates (ARMMAN), designed and implemented a mobile phone voice messaging service called mMitra. The overall Theory of Change aligned mobile messaging on key topics with prioritized health outcomes [7, 9]. mMitra aimed to help pregnant women improve self-care and uptake of key MNCH services during pregnancy through the first year of their children’s lives, primarily through behavior change. To date, the program has been implemented in nine states in India and has reached over 2 million women [9]. In the impact evaluation, a series of three questionnaires were administered to assess change in knowledge and uptake of practices known to improve maternal health outcomes in addition to acceptance of the mMitra intervention with a targeted focus on urban slums in Mumbai, India. This impact evaluation is registered with the ISRCTN (Trial# 88968111).

### Population and study design

Mumbai has 27 municipal wards, or administrative units, each with a population of roughly 800,000-900,000. Each ward is typically served by one maternity home and five or six health posts that provide pregnancy and infant health care services. Each ward appoints approximately 100 Angan Wadi Workers (AWWs) who make home visits, register pregnant women and motivate them to seek mother and child care. For this study, two such wards (F North and M East) were purposely selected for their large area, high population density and lack of prior exposure to mMitra. Researchers, who functioned independently of the AWWs, canvassed the wards to identify pregnant women for enrollment in the study.

Pregnant women in the two wards who spoke Hindi or Marathi – languages spoken by over 80% of the population in the city – were identified by research team members. The team members then followed up with eligible women to inform them of the study, obtain voluntary consent and enroll and assign them to the intervention or control group. Women without access to a mobile phone at home and not likely to be in Mumbai for 4–5 months during the pregnancy and after delivery (i.e., those planning to visit natal homes outside Mumbai for delivery, a common cultural practice in India) were not enrolled in the study.

The sample selection was single blind. At the time of enrollment, all women gave informed written consent prior to inclusion in the study and were not made aware of whether they were included in the intervention or the control group. Assignment to the intervention or control group was based on time of enrollment. For every 4 women consecutively enrolled in the study, the first 3 were assigned to the intervention group and the remaining woman was assigned to the control group. This approach was used in an attempt to ensure sufficient numbers of women were enrolled based on gestational age or pregnancy trimester to test the hypothesis that longer exposure to the messages would have an effect on outcomes (dose response) as the messages are designed to be delivered to coincide with the specific stage of the pregnancy. No other interventions were introduced alongside the messages. There was no known information contamination that was identified in the study. Women assigned to the intervention and control groups were followed by the investigators throughout their pregnancy until their infants turned one year of age. The intervention group received the mMitra voice messages in Hindi or Marathi, and the control group did not receive messages at all.

### mMITRA program and message design

One hundred and forty-five audio messages comprised the mMitra call package. The messages were designed by BabyCenter [10], adapted to local practices in partnership with ARMMAN and representatives from the Federation of Obstetric and Gynecological Societies of India and the Indian Academy of Pediatrics, and based on global (World Health Organization) and local (National Health Mission) guidelines. The audio messages were timed to the gestational age and developmental stage of the fetus and infant, respectively. They covered content relevant to women from when they were 6 weeks pregnant until their child reached 1 year of age. Specifically, this included nutrition, iron/folic acid supplementation, calcium supplements, development of the fetus, ANC reminders, anemia, rest during pregnancy, blood tests, HIV testing, sonography, danger signs during pregnancy, birth preparedness, identifying labor pains, cutting the umbilical cord/dressing the stump, family planning, sanitation and hygiene, breastfeeding practices, colostrum, and sex determination.

The messages were designed to be delivered two times per week during pregnancy. However, they were clustered at one message per day immediately post-partum for a week, and then reduced back to two messages per week until the child completed 1 year [9]. In case women missed messages, they could initiate a free call-back service within 2 days of the original call.

The messages, as well as their audio translations into Hindi and Marathi, were tested for appropriateness and cultural nuances with local health experts and through in field focus group studies. The voice and tone of the female recording artist were field-tested. The final message product was roughly 2 min long. The message began with a recognizable ‘jingle’ to alert the woman and her family; it ended by summarizing the key learning point.

### Sample size and power calculations

The sampling approach and sample size estimates were selected to ensure representativeness of the study population to the target population. The sample size was determined using a two-sample z proportion test. Sample size estimates were calculated for the primary outcome of interest: reduction of anemia during pregnancy. The District Level Household and Facility Survey 4 for urban poor in Maharashtra estimates the prevalence of anemia in pregnancy at 68.5% [11]. The sample size needed to detect a 10% reduction in anemia, assuming 30% attrition at an alpha of 0.05 and 80% power, was 392 per arm.

### Data collection

There were three rounds of data collection during this study: Pregnancy (baseline/Time 1); Post-partum (Time 2) and when the infant turned 1 year old (Time 3). There were no maternal health outcomes or indicators collected at Time 3. Satisfaction with the mMitra service for women receiving the service was assessed through a series of questions using a 5-point Likert scale. Data were collected by six female investigators who were part of the research team and went from house-to-house.

During the baseline period (Time 1), the investigators visited 100–110 homes per day in an attempt to enroll 4–5 women. Women who agreed to enroll in the study signed a consent form. They were then allocated either to the intervention or control group based on their time of enrollment. They then completed a survey administered orally by the investigator in either Hindi or Marathi – based on participant preference. The Time 1 Survey Questionnaire has been included as Supplementary Material S1-First interview mMitra. Their response was recorded on an Android tablet using the Kobo Collect platform. In the event of connectivity issues or low battery, the investigator filled out a paper-based form and entered the data into the tablet later that same day. At the end of each day, the investigators submitted their tablets to their supervisors. The supervisors reviewed the surveys and uploaded the data onto the central server. Every day, a data manager examined the uploaded data for completeness and consistency in responses. Any problems identified were discussed in daily morning briefings with the research team and addressed. The subsequent rounds of interviews were completed in the same manner.

In addition to the interviews, the investigators copied clinical information from the Mother and Child Health (MCH) card into the Kobo Collect platform. The MCH card is routinely issued to each woman at the health facility. The card contains information on services provided and clinical/laboratory findings (e.g., weight, blood pressure, hemoglobin level) of the woman and the child until the child reaches 1 year of age.

Information on maternal health knowledge and attitudes were collected at Times 1 and 2 through surveys. Data on maternal health outcomes were collected at Time 2 through surveys and MCH cards. In the intervention group, experience with mMitra was assessed at Time 2 through a semi-structured questionnaire. The Time 2 Survey Questionnaire has been included as Supplementary Material S2- Second interview mMitra.

In terms of study compliance, of the original 2016 women enrolled, 1760 women (87%) were reached for Time 2 interviews. 256 (13%) women could not be interviewed because some had gone to their mother’s house for delivery and had not returned home at the time of the interview held within 2 weeks after delivery (many of them had subsequently returned home and participated in Time 3 interviews when child health information was sought). Five women were reached but refused to be interviewed at Time 3.

### Outcome measures

The primary outcome of interest was anemia reduction. The data for this outcome were obtained at Time 2 and sourced from both the survey and triangulating with their MCH cards. It was selected for its clinical importance (e.g., low birth weight, premature birth, and maternal mortality) and prioritization by the Government of India for intervention. The hemoglobin level measured closest and prior to her date of delivery was used and obtained during Time 2. The data source was the MCH card. Hemoglobin levels greater than 110 g per Liter (g/L) were considered normal and indicative of the absence of anemia.

There were several additional outcomes of interest, including maternal health seeking knowledge, attitudes and practices. Most of the indicators related to maternal health seeking and care were included in the first two survey rounds (Times 1 and 2). Women’s responses to questions on proven ANC and post-natal care practices were compared at Times 1 and 2 and then in the intervention group versus control group.

### Statistical analysis

First, descriptive analyses were conducted to obtain a general sense of the data. Continuous data were compared between the intervention and control groups using two-sample t-test. When comparing continuous data from Times 1 and 2 across groups, the two-paired t-test was used. Categorical outcomes were assessed using chi-square tests. For the outcomes data, simple and binary logistic regressions were conducted. Intention to treat and per protocol analyses were conducted and compared. All data were analyzed using SPSS version 18.0.

### Ethics approval

The study was approved by the Foundation for Research in Health Systems’ Institutional Review Board under Protocol Number HHS00009235. Written informed consent was obtained from all participants.

## Results

**Fig. 1:**
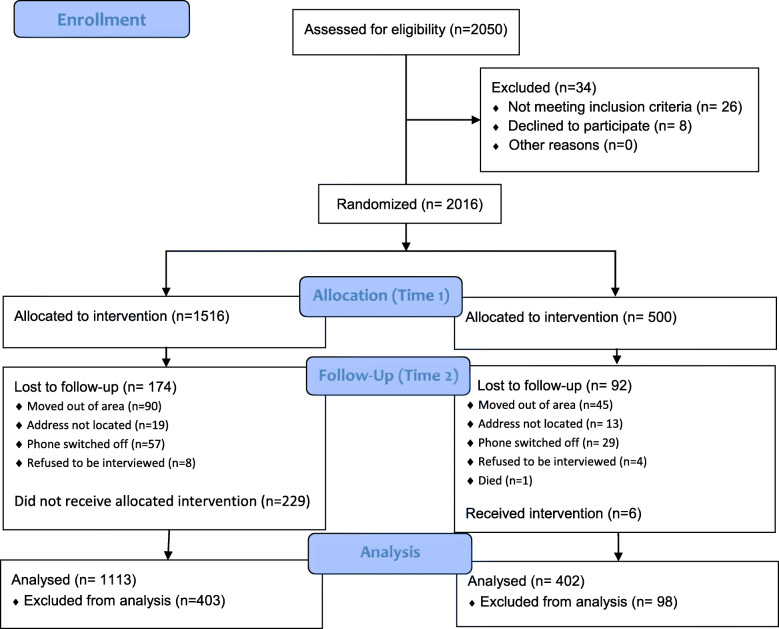
Flow Diagram of women enrolled in the mMitra pseudo-randomized controlled trial from Time 1 to Analysis informed by CONSORT Guidelines

The evaluation of mMitra took place from June 2015 to January 2017. Over 23,500 households were visited and 2050 pregnant women were identified as eligible. A total of 2016 pregnant women were enrolled in the study. Of those, 1516 were allocated to the intervention group and 500 to the control group (Fig. 1).

### Timeline

Enrollment and baseline surveys (Time 1) lasted from June–October 2015, ending when the requisite numbers of women had been enrolled in the intervention and control groups. The post-partum surveys (Time 2) started in November 2015 and ended in March 2016 when all women enrolled had delivered their babies.

### Characteristics of the study population

There were 2016 pregnant women enrolled in the study. At baseline (Time 1), the 1516 women in the intervention group and 500 women in the control group were similar in age, parity, education, family type, and membership in a social community [12]. However, women in the intervention group were more likely to be employed, own a mobile phone, listen to a radio, read the newspaper, and have literate and employed husbands [12]. This changes at Time 2 when conducting per protocol analyses: women in the intervention group were more likely to be older (*p* = 0.001) and watch TV (*p* = 0.013) yet have no difference in employment status, newspaper reading, or their husband’s literacy or employment status as compared to controls (Table 1).

**Table 1:** Socio-demographic Characteristics of Intervention and Control Groups at Time 2

Variable	Intervention N (%)	Control N (%)	***P*** Value
**Number of women**	1113	402	
**Variable**			
Median age of women	25 (SD 3.9)	24 (SD 4.1)	0.001*
Women’s age < 25 years	60.7	64.2	0.2161
First time pregnant	30.5	31.8	0.6286
Women’s education > 10 years	39.4	42.8	0.2336
Woman employed	15.5	10.0	0.0065*
Living as Nuclear family	51.8	49.0	0.3358
Has older woman living in the house	41.1	44.5	0.2365
Belong to SC/ ST ^1^ group	18.1	17.2	0.6864
Watches TV	85.6	80.3	0.0125*
Listens to radio	15.4	11.2	0.0391*
Woman owns mobile phone	87.6	81.1	0.0013*
Reads newspaper	35.7	31.0	0.0892
Husband literate	81.1	84.8	0.0973
Husband employed	98.4	97.8	0.4328

*p < 0.05, ^1^Schedule castes (SC) and Schedule Tribes (ST) are a set of communities identified in Indian constitution as being socially disadvantaged and therefore needing special development assistance

Of the women enrolled, 1750 (87%) were reached at Time 2. There were 174 women (11.4%) lost to follow-up in the intervention group compared to 92 (18.4%) in the control group. The reasons for attrition included moving out of the area, addresses not locatable, mobile phones switched off or women refusing to be interviewed (Fig. 1). Unfortunately, one woman in the control group died during delivery. Also, at Time 2, 16 (3.9%) of the 418 women in the control group reported receiving mMitra calls, and 229 (17%) of the 1342 women in the intervention group reported never receiving mMitra phone calls. An NGO working in the neighboring area had inadvertently enlisted a few women in the control group to receive mMitra messages. The women in the intervention group who reported that they did not receive mMitra calls was due to Do-not-Disturb (DND) option being activated on their phones so the messages could not be delivered. Some women in the intervention group reported not receiving messages though we could not verify the reasons. According to per protocol analyses, the data for these women were discarded, resulting in the data of 1515 women being analyzed (intervention *n* = 1113, control *n* = 392).

### MCH cards

Women in both groups did equally well in retaining their MCH cards. There were 1734 women (87.5% in the intervention group, 81.6% in the control group) who showed their MCH cards at Time 1. At that time, 12% of women in their first trimester reported that they were yet to receive their cards, and 19% had the cards but refused to show them to the investigators. More women in both groups at Time 2 showed their cards to investigators (93.4% in the intervention group, 94.8% in the control group). However, there was a small but significant difference in the recording of health information in the MCH cards, with women in the intervention group having more complete cards as compared to controls for the number of ANC visits completed, weight and blood pressure measurements during ANC visits, and receiving a tetanus shot.

There were also discrepancies found between women’s self-reports (via the surveys) and documentation in the MCH cards for both groups. For example, over 90% of women self-reported making at least 3 ANC visits but only 20% of cards had a record of three or more ANC visits. Similarly, 96% of women self-reported receiving a tetanus toxoid injection while only 25% of cards had recorded administration of a tetanus toxoid injection. None of the MCH cards showed women being provided or prescribed calcium tablets, though over 67% of women reported taking calcium tablets during pregnancy.

### Impact on maternal health practices

The impact of mMitra on maternal care practices was compared between intervention and control groups using data collected at Time 2 on 22 maternal practice indicators (Table 2). The practice levels at the baseline ranged from 28% (early registration of pregnancy) to 99% (took rest/nap in the afternoon), which showed consistency between practice and knowledge (see below). The intervention group performed significantly better on the following 4 practice indicators: receiving the tetanus toxoid injection (OR: 1.6, 95% Confidence Interval (CI): 1.05–2.4, *p* = 0.028), consulting a doctor if spotting or bleeding (OR: 1.72, 95%CI: 1.07–2.75, *p* = 0.025), saving money for delivery expenses (OR: 1.79, 95%CI: 1.38–2.33, *p* = 0.0001), and delivering in hospital (OR: 2.5, 95%CI: 1.49–4.35, *p* = 0.001). While not statistically significant (*p* = 0.077), women in the intervention group had 28.5% increased odds of registering their pregnancy in the first trimester as compared to controls.

**Table 2:** Impact of mMitra on maternal practices at Time 2 (adjusted analyses)

	Respondent category (Intervention/Control)	***P*** value
Registered pregnancy in 1st trimester	1.285 (0.973–1.696)	0.077
ANC Visits 3 or more	1.508 (0.797–2.853)	0.207
Took TT injection	1.596 (1.053–2.4128)	0.028*
Took Calcium	1.218 (0.943–1.574)	0.131
Consumed 100+ IFA tablets	0.905 (0.710–1.153)	0.419
Ate more food during pregnancy	0.854 (0.676–1.079)	0.187
Ate Green vegetables	0.868 (0.417–1.807)	0.706
Ate Fruits during pregnancy	1.006 (0.773–1.310)	0.962
Drank Milk during pregnancy	1.070 (0.842–1.360)	0.581
Ate pulses and beans during pregnancy	0.976 (0.720–1.322)	0.875
Took nap/rest, regularly during pregnancy	0.690 (0.544–0.875)	0.002*
Husband accompanied for ANC (regularly /mostly)	0.870 (0.686–1.104)	0.253
Consulted doctor if they had swelling on hands and feet	1.005 (0.780–1.294)	0.972
Consulted doctor if suffered from tiredness	0.997 (0.775–1.282)	0.979
Consulted doctor in case of spotting/bleeding	1.715 (1.070–2.748)	0.025*
Saved money for delivery expenses	1.790 (1.375–2.329)	0.0001*
Husbands not smoking at home/among smokers	0.475 (0.129–1.755)	0.264
Delivered in hospital	2.543 (1.488–4.348)	0.001*
Women discussed FP method with husband	0.816 (0.640–1.040)	0.101
Women adopted FP method after delivery	0.784 (0.577–1.065)	0.119
Mother fed colostrum to the baby	1.269 (0.898–1.794)	0.177
Delivery by Skilled birth attendant at home deliveries	0.457 (0.229–0.913)	0.027*

*p < 0.05

By contrast, the control group performed significantly better than the intervention group on 2 practices indicators: resting regularly during pregnancy (OR: 0.7, 95%CI: 0.54–0.88, *p* = 0.002) and having at-home deliveries attended by a skilled birth attendant (OR: 0.46, 95%CI: 0.23–0.91, *p* = 0.027).

The groups performed similarly for the remaining practices during pregnancy, especially those related to anemia control, such as consuming iron and folic acid supplements, eating more frequently, eating nutritious food during pregnancy, and consulting the doctor for symptoms like tiredness (when experienced).

### Impact on clinical maternal health outcome of Anemia

Clinical data on hemoglobin levels were only available for 24.3% of women in the control group and 33.8% of women in the intervention group. Subsequently, anemia status at or near the time of delivery, based on hemoglobin levels, could not be assessed.

### Impact on maternal care knowledge

In the study population, correct responses to various knowledge questions at the baseline, ranged from 13 to 98%. The lowest performing knowledge question was “During which month of pregnancy should a woman first see a doctor? (first trimester: 13%). On nutrition related questions the responses were equivocal, like: “As compared to her usual intake, should woman eat more / less/usual quantity of food during pregnancy? (More = 46%) and “which tablets should women take for supplementation? (Iron =56%). However, near universal awareness was recorded to the question “pregnant women need to take more rest/nap in the afternoon (92%).

For 13 of the 15 knowledge indicators compared at Times 1 and 2, there were improvements within both groups. However, one knowledge indicator was significantly increased among women in the intervention group: women should see a doctor in the first trimester of pregnancy. When comparing knowledge at Time 2 in the intervention group versus controls, adjusted analyses indicated that women in the intervention group had 2.13 times increased odds of agreeing that medical support is required during pregnancy (95%CI: 1.17–3.86, *p* = 0.013). There were no indications of differential knowledge on the other health topics including other reasons for medical support during pregnancy, nutrition and supplements, and birth spacing. Additionally, there was no discernable association between background demographic characteristics and knowledge levels.

### Women’s experience with mMitra messages

Of the women in the intervention group at Time 2, 975 (88%) report receiving the mMitra calls. For the remaining 12%, system data on call logs indicate that calls were being sent to the designated number, but nearly 50% were not answered and the other half were answered by family members (not the woman).

Close to 43% of women reported listening to the calls by themselves. Roughly 79% of the women who reported receiving the calls said that they often to always listened to the entire message. Close to 91% of women were satisfied to very satisfied with the mMitra calls, and 92% found that the calls were useful. Only 28.7% knew about the “missed call” function, and 19.2% reported using it.

Approximately two thirds of respondents reported discussing information contained in messages with family members and friends (60% with other family members, 61% with mother-in-law, 65.2% with friends). Most women (86%) reported discussing the information with their husband. Over 60% of women reported that mMitra had improved their health awareness, and by sharing that information with family members they felt comfortable in seeking assistance in daily chores and in taking care of themselves. However, only one third of women said that they felt comfortable discussing pregnancy-related problems with the health staff.

## Discussion

This study provides evidence that tailored mobile phone-based voice messages can improve key maternal behaviors and maternal knowledge among women in low-resource settings. Women exposed to mMitra messages improved adoption of both home- and health facility-based practices that can positively impact maternal and child health outcomes.

### Improvements in health-seeking behavior

Exposure to a voice messaging service, like mMitra, could potentially improve access to and use of recommended services for routine and high-risk pregnancies thereby improving maternal outcomes. For example, women in the intervention group were more likely to receive their tetanus toxoid injection and deliver in hospital. There was a trend towards registering their pregnancy in the first trimester, and there were also slightly more women who completed three or more ANC visits. These findings are in line with previous reports where mobile phone text-based messages increased attendance of ANC visits in low and middle-income countries [13–17], and there are recent studies that report that mobile phone text-based interventions improved skilled birth attendance and uptake of facility-based maternal care [1, 13, 15].

While it is reasonable to assume change in knowledge levels will lead to behavior change and practices, additional research is needed to map out the pathways through which mHealth interventions contribute to behavior change for MNCH, and to what degree those depend on change in knowledge alone as well as exposure to the intervention. Furthermore, since voice messages are accessible to women of any literacy level and low techno-literacy levels, it will be useful to compare voice and text on ‘softer’ outcomes such as emotional connection, relatability and tailoring.

### Anemia

In this study, we attempted to directly assess the impact of the intervention on the maternal health outcome of anemia. The relevant biomarker, hemoglobin levels, was recorded in the MCH cards. Although we had access to MCH cards of over 90% of women in both intervention and control groups, we found only 20–30% of cards containing data on hemoglobin levels, limiting the sample size needed to detect a statistically significant difference in the outcome of interest and minimizing our confidence in the data. The fact that we observed significant increases in the information recorded on the number of ANC visits and patient level data among women in the intervention cohort was an important positive result of the intervention.

### Empowerment during pregnancy

Overall, significantly fewer women in the intervention group reported taking rest and/or taking naps compared to the control group. This could be because fewer women in the control group were employed and therefore were more likely to have an opportunity to take naps/rest regularly. Nevertheless, women in the intervention group appeared to take a more active control of their health during pregnancy and prepare for delivery, including saving money for delivery expenses and consulting a doctor in case of spotting/bleeding.

Women receiving the mMitra messages also shared and discussed the information they received with others, especially family members, and felt more confident asking for assistance around the house. This is especially important in communities where behavior change depends on buy-in and support within an individual’s social-ecosystem [18, 19], and it underscores the potential of tailored voice messages to create shared emotional connections to the pregnancy and to strengthen such social support systems. This is valuable for promoting behavior change that improves MNCH outcomes. Future studies could test whether sharing such information is directly correlated with increased adoption of maternal care practices.

### Knowledge

The study did not show many significant changes in increased knowledge on maternal care topics in the intervention group. Changes in knowledge may not have been detected as not all knowledge indicators measured in this study were directly correlated to specific messages or practices. Instead the study aimed to gain a general understanding of knowledge on a broad set of topics related to maternal, newborn and child care among poor, urban women. Knowledge levels did increase in both the control and intervention groups as pregnancy continued suggesting that women had access to other quality sources of information (peers, health workers, TV, radio media etc.) possibly due to being in urban settings. As such, it would be difficult in this context to attribute any increase in knowledge to the mMitra intervention alone. Furthermore, the level of knowledge on some topics was already high and two-thirds of women in both control and intervention groups had been pregnant before, potentially making them extremely familiar with these topics already.

### Limitations

Our study relied primarily on self-reported data obtained by survey from women, which could be subject to recall bias and even inaccurate or false reporting by women. Loss to follow up and compliance differences in the control and intervention groups led to reductions in data that was included in the analyses. However, this was anticipated in the design of the study as it is known that many women will go to their maternal homes for delivery. The sample calculation was conducted to account for this. While clinical records like MCH cards can provide valuable information on actual ANC services received and help overcome self-reporting bias, issues related to data quality in official health records can be difficult to overcome, especially when clinics are crowded, and health care providers are very busy providing service or women forget or lose their cards. Relatedly, there were also discrepancies between data on the MCH cards and what was reported by women. There was a missed opportunity to obtain and use system data, which is objective and not subject to the same biases as self-reported data. Another limitation of the study was the inability for the data to be linked to specific behavioral practices as there was not a one-to-one matching of messages to health outcomes or indicators. In addition, several mMitra messages address the same outcome, and single messages can also be linked to multiple outcomes and indicators.

Separately, the contamination of the control group was due to mMitra implementations outside of the impact evaluation being conducted in nearby areas or women in the intervention group informing their friends of the mMitra messages. Lastly, the randomization method is susceptible to misclassification. Therefore, the study may not have been as well balanced as a traditional randomized controlled trial. Unfortunately, intention to treat analyses could not be conducted nor compared to the per protocol analyses as part of sensitivity analyses.

## Conclusion

This study adds to the growing body of evidence on the impact of mHealth interventions on behaviors proven to improve MNCH outcomes. To our knowledge, this study is one of the first voice interventions for MNCH to be evaluated systematically by a pseudo-randomized controlled trial design in a low–resource setting. Our findings suggest that the voice messages may make women more inclined to act on information, even though big differences in knowledge were not apparent. Further research is recommended to assess the relationship between changes in knowledge and behavior as well as to compare voice and text message interventions ‘head-to-head’ on MNCH outcomes. Such research should also systematically explore the differential impacts of tailored voice messages compared to text messages on behavior and practice changes among women’s family members.

## Supplementary information

**Additional file 1 Supplement 1**. Time 1 (Baseline) Survey. Questionnaire FIRST INTERVIEW: Impact Evaluation of mMitra.

**Additional file 2 Supplement 2.** Time 2 (Post-Partum) Survey. Questionnaire SECOND INTERVIEW: Impact Evaluation of mMitra.

## Data Availability

The datasets used and/or analyzed during the current study are available from the corresponding author on reasonable request.

## Abbreviations

ANC: Antenatal care
LMIC: Low- and middle- income country
MNCH: Maternal newborn and child health
MCH: Maternal child health
mHealth: Mobile health
