# Supplementary material for: Effects of an mHealth voice message service (mMitra) on maternal health knowledge and practices of low-income women in India: findings from a pseudo-randomized controlled trial
Source: BMC Public Health. 2020 Jun 1;20:820. doi: 10.1186/s12889-020-08965-2 (PMC7268375; doi:10.1186/s12889-020-08965-2)
Supplement: Supplementary file 1 — Additional file 1 Supplement 1. Time 1 (Baseline) Survey. Questionnaire FIRST INTERVIEW: Impact Evaluation of mMitra. [file 12889_2020_8965_MOESM1_ESM.docx]

**Supplement 1. Time 1 (Baseline) Survey**

*For Research Purposes Only*

**Questionnaire FIRST INTERVIEW: Impact Evaluation of mMitra**

| **Sr. No.** | **Questions** | **Response** | **Code** |
| --- | --- | --- | --- |
| 1 | ***Investigator code*** |  |  |
|  | ***1. Identification Details*** |  |  |
| 1.1 | Date of the interview  **(DO NOT ASK, PLEASE FILL IT ON YOUR OWN)**  **साक्षात्कारकी तिथि** | dd/mm/yyyy |  |
| 1.2 | Serial number of the Respondent  **(DO NOT ASK, PLEASE FILL IT ON YOUR OWN)** |  |  |
| 1.3 | Name of the Respondent  **उत्तरदेनेवाले**का नाम |  |  |
| 1.4 | Is this your usual place of residence or are you visiting? | Usual Resident  Visitor | 1 GO TO 1.6  2 |
| 1.5 | If you are visiting, how long will you stay here? Will you stay here till you deliver and a baby becomes one year? | No, will stay only for a short time  Yes, will be here till the child turns 12 month old | 1 (STOP 1)  2 |
| 1.6 | Does anyone in your household have a mobile phone? | Yes  No | 1  2 (STOP 2) |
| 1.7 | Usually, whose mobile phone do you use? | Self  Husband  Other family members | 1  2  3 |
| 1.8 | Have you heard of mMitra Voice call service for pregnant women? (Show mMitra brochure to confirm) | Yes  No | 1  2 GO TO 1.11 |
| 1.9 | Have you been enrolled for this service during this pregnancy? | Yes  No | 1  2 GO TO 1.11 |
| 1.10 | Have you started receiving the voice calls? | Yes  No | 1 (STOP 3)  2 |
| Now let me inform you about the study I mentioned in the beginning. Read the consent form. | | | |
| 1.11 | Will you be willing to participate in the study? | Yes  No | 1  2 (STOP 4) |
| 1.12 | Name of head of the household     घरके मुखियाका नाम | A) First_________________  B) Middle_________________  C) Surname_________________ |  |
| 1.13 | Address of the Household    घरका पता | A) HH#_________________  B) Landmark_________________  C) Locality_________________ |  |
| 1.14 | Respondent’s (mobile) Phone number  **उत्तरदेनेवाले**का मोबाइल फ़ोन नंबर | _________________ |  |
| 1.15 | Other member’s (mobile) phone number  **अन्यसदस्यका (मोबाइल) फोन नंबर** | _________________ |  |
| 1.16 | Gestation month of the respondent  **उत्तर देनेवाले की** गर्भावस्थाका महीना | ________ |  |
| 1.17 | Last Menstrual date (est)  आखरी माहवारी की तारीख | dd/mm/yyyy |  |
| 1.18 | Name of the Interviewer  **साक्षात्कारकर्ता का नाम** | ______________________________ |  |
| *Now I am going to ask you some questions about your household.*  अब में आपसे आपके घर के बारेमें कुछ प्रश्न पूछना चाहती हूँ . | | | |
| 2.1 | What is your relationship to the head of the household?    आपके घर के मुखिया से आपका क्या रिश्ता है? | Husband  Father-in-law  Mother-in-law  Brother-in-law  Other (specify)______________ | 1  2  3  4  8 |
| 2.2 | What is your religion?     आपका धर्म क्या है? | Hindu  Muslim  Christian  Buddhist  Other (specify)______________  Don't know/ Can't say | 1  2  3  4  8  999 |
| 2.3 | What is your caste?  Is this a scheduled caste, a scheduled tribe, other backward class, or none of these?  (ASK RESPONDENTS OF ALL RELIGIONS)  आपकी जाती क्या है? क्या ये जाती – अनुसूचित जाती है, अनुसूचित जनजाति है, अन्य पिछड़ी जाती याकोई अन्य जाती है? | Scheduled caste  Scheduled tribe  Other backward caste  Other (specify)______________  Not reported | 1  2  3  8  999 |
| 2.4 | How many persons usually live in this household?  आपके घर में आमतौर पर कितने लोग रहते हैं? | __________________________ |  |
| 2.5 | Your family residing in this household is nuclear or joint?  (A FAMILY WITH RESPONDENT, HER HUSBAND AND CHILDREN IS NUCLEAR)  क्या इस परिवार में सिर्फ आप, आपके पति और बच्चे रहते हैं, या साथ में अन्य सदस्य भी रहते हैं?  (Nuclear family - सिर्फ **उत्तरदेने वाली, उनके पति और बच्चे)** | Joint  Nuclear | 1  2 |
| 2.6 | Does your mother-in-law usually live in this household?  **क्या आपकी सास** आमतौर पर **इसी घरमें रहती है ?** | Yes  No | 1  2 |
| 2.7 | Is there any other female member who is older than you, usually living in this household?  **क्या इस घर में आप से उम्र में बड़ी कोई और महिला** सदस्य **भी रहती है ?** | Yes  No | 1  2 |
| 2.8 | Currently is any other household member pregnant?  क्या अभी आपके घर में कोई **और** भी गर्भवती हैं? | Yes  No | 1  2 |
| 2.9 | Type of house  (DO NOT ASK, OBSERVE AND RECORD THE MATERIAL USED)  मकान का प्रकार | *Kaccha*  Semi-*pucca*  *Pucca* | 1  2  3 |
| 2.10 | What type of fuel does your household mainly use for cooking?    आपके घर में खाना बनाने के लिए आमतौर पर किस प्रकार का ईंधन इस्तमाल कियाजाता है? | LPG  Kerosene  Coal  Wood  Electricity (heater/induction)  Other (specify)______________ | 1  2  3  4  5  8 |
| 2.11 | How many rooms are there in your house?  आपके घर में कितने कमरे हैं? | ______________________________ |  |
| 2.12 | Does your household have a ration card?  क्या आपके घर में राशन कार्ड है? | Yes  No | 1  2  GO TO 2.14 |
| 2.13 | What is the type of your ration card?    आपका राशन कार्ड कौन से रंग का है? | White  Orange  Yellow  Other (specify)______________ | 1  2  3  8 |
| 2.14 | Do you have a TV in the house?  क्या आपके घर में टी वी है? | Yes  No |  |
| 2.15 | Do you have radio in the house?  (RADIO IN ANY FORM)  क्या आपके घर में रेडियो है? | Yes  No |  |
| 2.16 | What is your source of drinking water?  आपके पीने के पानी कहाँ से लेते हैं? | Tap at home  Community tap  Water tanker  Bottle water  Other | 1  2  3  4  8 |
| 2.17 | What type of latrine facility do you use?  आप किस तरह का शौचालय इस्तमाल करते हैं? | Latrine at home  Common latrine facility – Paid  Common latrine facility – Unpaid  Paid municipal facility  Open space | 1  2  3  4  5 |

| 3.0 | **3. Individual Information** | ***Now, I am going to ask you some questions about yourself and your husband*** |  | |
| --- | --- | --- | --- | --- |
| 3.1 | Please tell me your husband’s name.  आपके पति का नाम क्या है? | ______________________________ |  | |
| 3.2 | How old are you?  आपकी उम्र कितनी है? | _________ Years |  | |
| 3.3 | How old is your husband?  आपके पति की उम्र कितनी है? | _________ Years |  | |
| 3.4 | How long have you been living in Mumbai?  आप मुंबई में कितने समय से रह रही हैं? | Less than 1 year  1-2 years  2-5years  5years and above  Since birth | 1  2  3  4  5 | |
| 3.5 | What is your mother tongue?  आपकी मातृभाषा क्या है? | Marathi  Hindi  Other | 1  2  3 | |
| 3.6 | What is the highest educational standard you have completed?  आपने कहाँ तक पढ़ाई की है? | No Formal education  Std. 1-4  Std. 5-7  Std. 8-9  Std. 10-11  Std. 12  Degree/Diploma | 1  2  3  4  5 Go to 3.8  6  7 | |
| 3.7 | (IF RESPONSE CODE 1 OR 2, THEN ASK)  Can you read and write?  क्या आप लिखना और पढ़ना जानती हैं? | Yes  No | 1  2 (Go to 3.9) | |
| 3.8 | How often do you read a newspaper or magazine?    **आप कितनी बार अखबार "ya" पत्रिका पढ़ती हैं?** | Almost every day  Once a week  Occasionally/Rarely  Not at all | 1  2  3  4 | |
| 3.9 | How often do you watch television?  **आप कितनी बार टीवी देखती हैं ?** | Almost every day  Once a week  Occasionally/Rarely  Not at all | 1  2  3  4 | |
| 3.10 | How often do you listen to the radio?    **आप कितनी बार रेडियो सुनती हैं ?** | Almost every day  Once a week  Occasionally/Rarely  Not at all | 1  2  3  4 | |
| 3.11 | Do you own a mobile phone?  **क्याआपके पास अपना मोबाइल फोन है ?** | Yes  No | 1  2 | |
| 3.12 | What kind of phone is it?  आपका फ़ोन किस तरह का है? | Basic  Smart | 1  2 | |
| 3.13 | Which functions of the phone do you use?  **MULTIPLE OPTIONS**  **(READ OUT ALL OPTIONS)**  आप फ़ोन का इस्तमाल किस-किस काम के लिए करती हैं? | 1)Calling ca  2) SMS  3) Camera  4) Music  5) Internet  6) Social Media  7) Email  8) Mobile banking  9) Others (Specify) _________ | Yes  1  1  1  1  1  1  1  1  1 | No  2  2  2  2  2  2  2  2  2 |
| 3.14 | How often do you use the mobile phone?  **आप** फ़ोन का इस्तमाल **कितनी बार** करती हैं**?** | Almost every day  Once a week  Occasionally/Rarely  Not at all | 1  2  3  4 | |
| 3.15 | Apart from your own household chores, are you currently engaged in any paid work?  **अपनेघरकेकामकेअलावा, क्या आप आज कल पैसा कमाने के लिए भी कोई काम करती हैं?** | Yes  No | 1  2 | |
| 3.16 | What type of paid work were you engaged in last one month?  **पिछले एक महीने में आपने पैसे कमाने के लिए किसी तरह का काम किया है?** | Unskilled Laborer/Housemaid  Skilled worker  Self Employed  Office work  Teacher  Professional | 1  2  3  4  5  6 | |
| 3.17 | What is the highest educational standard your husband has completed?  आपके पति कहाँ तक पढ़े हुए हैं? | No Formal education  Std. 1-4  Std. 5-7  Std. 8-9  Std. 10-11  Std. 12  Degree/Diploma | 1  2  3  4  5  6  7 | |
| 3.18 | Is he currently employed?  क्या आपके पति आज कल कहीं काम कर रहे हैं? | Yes  No | 1  2 Go to 3.20 | |
| 3.19 | What type of work he was engaged in last one month?  **पिछले एक महीने में** आपके पति**ने किस तरह का काम किया है?** | Unskilled Laborer  Skilled worker  Self Employed  Office work  Teacher  Professional | 1  2  3  4  5  6 | |
| 3.20 | Approximately how much is your household’s monthly income?  **आपके घर की लगभग एक महीने की कितनी कमाई है ?** | ____________ Rs.  No responses | 999 | |
|  | ***4. Respondent’s obstetric History*** | *Now, I am going to ask you about your pregnancies and children.* |  | |
| 4.1 | What was your age when you got married for the first time?  जब आपका विवाह हुआ था तब आपकीउम्र कितनी थी? | ____________ Years |  | |
| 4.2 | Is this your first pregnancy?  क्याआप पहली बार गर्भवती हुई हैं? | Yes  No | 1 Go to 4.6  2 | |
| 4.3 | What was your age (completed) when you became pregnant for the first time?  जब आप सबसे पहली बा रगर्भवती हुई तब आपकी उम्र कितनी थी? | ____________ Years  No response | 999 | |
| 4.4 | How many children did you give birth to?  **आपने कुल कितने** बच्चों कोजन्म दिया? | 1)Sons_____________  2) Daughter |  | |
| 4.5 | Out of them, how many are currently living?  (CHECK THAT THE NUMBER GIVEN IN 4.5 SHOULD NOT BE MORE THAN 4.4)  अभी आपके कितने बच्चे हैं? | 1)Sons_____________  2) Daughter |  | |
| 4.6 | Have you ever had any stillbirths, abortions, miscarriages?  जीवित जन्मों के अलावा, क्या किसी भी गर्भावस्थाके दौरान आपने गर्भपात कराया, या बच्चा गिर गया या मृत पैदा हुआ? | Yes  No | 1  2 Go TO 4.8 | |
| 4.7 | How many?  कितने? | 1)Still births: ______________    2)Abortions:______________    3) Miscarriages: __________ |  | |
|  |  |  |  |  |
|  |  |  |  |  |
| 4.8 | What is the order of this (current) pregnancy?  (PLEASE ADD NUMBERS IN 4.4 + 4.7+ 1)  ये आपका कौन सा गर्भ है? | _____________ | If the order of pregnancy is 1, Go to 4.10 | |
| 4.9 | How old is your youngest child?  **आपके सब से छोटे बच्चे कीउम्र क्या है?** | Less than 6 months  6months – 1year  1year -3years  3 years and above | 1  2  3  4 | |
| 4.10 | When you became pregnant this time did you want this pregnancy, did you not want this pregnancy or you/your husband were using family planning method and it failed?  जब आप इसबार गर्भवती हुईं, तब क्याआप चाहती थीं, या नहीं चाहती थीं, या आप और आपके पति गर्भनिरोधक का इस्तमाल कर रहे थे और चूक हो गयी? | Planned  Unplanned  Due to method failure | 1  2  3 | |
|  |  |  |  |  |
|  |  |  |  |  |
| 5.0 | *Now, I am going to ask you some questions about relationship you share with your husband and other household members.*  अब मैंआपसेकुछसवालआपकेघरवालोंकेसाथआपकेकैसेसम्बन्धहैं, उसकेबारेमेंपूछूँगी . | | | |
| 5.1 | Do you currently use any of these?  (READ OUT ALL OPTIONS)  क्याआप इनमें से कुछ भी इस्तमाल करती हैं? | A) Chews tobacco  B) Uses masher  C) Smokes  D) None | Yes  1  1  1  1 | No  2  2  2  2 |
| 5.2 | Does your husband smoke cigarettes or bidis?  **क्या आपके पति सिगरेट या बीड़ी पीते हैं?** | Yes  No | 1  2 Go to 5.4 | |
| 5.3 | Does he smoke at home or outside?  **क्या आपके पति सिगरेट या बीड़ी घर पर पीते हैं या फिर बाहर?** | Home  Outside | 1  2 | |
| 5.4 | Does your husband consume alcohol?  (PROBE)  **क्या आपके पति शराब पीते हैं?** | Yes  No | 1  2 Go to 5.6 | |
| 5.5 | How frequently does he consume alcohol?     वह कितनी बार पीते हैं? | Almost every day  Once a week  Less than once a week  Rarely  Can't say /NR | 1  2  3  4  999 | |
|  |  |  |  |  |
|  |  |  |  |  |
|  |  |  |  |  |
|  |  |  |  |  |
| 5.6 | How frequently does your husband travel outside city for work?  (PROBE)  **आपके पति काम के सिलसिले में कितनी बार शहर से बाहर जाते हैं?** | Most of the time  Once a week  Once a month  Rarely  Never | 1  2  3  4  5 | |
| 5.7 | When you are unwell, does your husband go with you to doctor?    जब आपकी तबियत ख़राब होती है तो क्या आपके पति आपके साथ डॉक्टर के पास जाते हैं? | Yes, most of the times  Yes, if it is serious  No, because he is busy  Not at all | 1  2  3  4 | |
|  |  |  |  |  |
|  |  |  |  |  |
|  |  |  |  |  |
| 5.8 | Do you share your health problems, even the smaller ones, with your husband?  **क्या आप आपने पति से अपनी स्वास्थय सम्बंधित परेशानियां के बारेमें, छोटी से छोटी बात भी करती हैं?** | Yes  No | 1  2 | |
| 5.9 | When you are unwell, and your husband is unavailable, who goes with you to doctor?  जब आपकी तबियत ख़राब होती है और अगर आपके पति मौजूद नहीं हैं तो आपके साथ डॉक्टर के पास कौन जाता है? | No one  Other household members  Other family members/relatives  Friends/Neighbours | 1  2  3  4 | |
|  |  |  |  |  |
|  |  |  |  |  |
|  |  |  |  |  |
| 5.10 | Does your husband ever beat you?  क्या आपके पति ने आपको कभी मारा है/या आप पर हाथ उठाया है? | Yes  No | 1  2  Go to Section 6 | |
| 5.11 | Has he beaten you in the last 3 months?  **क्या आपके पति ने आपको पिछले ३ महीनों में**  मारा **/ हाथ उठाया है?** | Yes  No | 1  2 | |
|  | ***6. Awareness of Maternal Health Care*** | | | |
| There are many customs and practices regarding diet, rest, health care to be followed during pregnancy. Now I am going to ask you some questions regarding your beliefs in these.  गर्भावस्था के दौरान देख भाल के बहुत सारे रीती-रिवाज़ होते हैं, अब मैं आपसे उसके बारे में सवाल पूछूंगी\| | | | | |
| 6.1 | Pregnancy and child birth is a natural phenomenon. Do you think any medical help is required?  गर्भावस्था और बच्चेका जन्म प्राकृतिक/कुदरती है. क्या आपको लगता है की इस में डॉक्टर की मददकी ज़रूरत है? | Yes  No  Can't say | 1  2  999 | |
| 6.2 | If a woman does not have any health problem during pregnancy, do you think then also she should see a doctor?  अगर किसी महिला को गर्भावस्था में कोई तकलीफ नहीं है, तो क्या फिर भी उसे डॉक्टर को दिखाना चाहिए? | Yes  No  Can't say | 1  2  999 | |
|  |  |  |  |  |
|  |  |  |  |  |
| 6.3 | During which month of pregnancy should a woman first see a doctor?  गर्भावस्था के किस महीने में महिला को डॉक्टर को पहली बार दिखाना चाहिए? | First Trimester  Any other responses  Can't say/DK | 1  2  999 | |
| 6.4 | How frequently a woman should see a doctor during her pregnancy?  गर्भावस्था में महिला को डॉक्टर के पास जाँच के लिए कितनी बार जाना चाहिए? | 1 time  2 times  3 times  4 times  As many times as needed  Can't say/DK | 1  2  3  4  5  999 | |
| 6.5 | Do you think it is necessary for a pregnant woman to take some tablets for supplementation of nutrients?  क्या गर्भावस्था के दौरान ताकत की गोलियां लेना ज़रूरी है ? | Yes  No  Can't say | 1  2  9 99 Go to 6.7 | |
| 6.6 | Do you know which tablets women have to take for supplementation?  क्या आप बता सकती हैं कि ताकतकी कौन कौन सी गोलियां लेनी चाहिए? | Iron  Calcium  Other  DK | 1  2  3  999 | |
| 6.7 | Do you know about an injection pregnant woman needs to take to protect from tetanus?  क्या आपको सुई (इंजेक्शन) के बारे में पता है जो महिला को टेटनस से बचाव के लिए लेना चाहिए ? | Yes  No  DK | 1  2  999 | |
| 6.8 | As compared to her usual intake, do you think a pregnant woman should eat more / less/usual quantity of food?  गर्भावस्था में क्या महिला को खाना पहले से ज़्यादा खाना चाहिए, काम खाना चाहिए या जितना खाती थी उतना ही खाना चाहिए? | More food  Less food/ Same as usual  Can't say | 1  2  999 | |
| 6.9 | Do you think a pregnant woman should include some nutritious items especially in her diet?  क्या गर्भवती महिला को खासतौर पर पौष्टिक/ताकत वाली चीज़ें खानी चाहिए? | Yes  No  Can't say | 1  2 Go to 6.11  999 | |
|  |  |  |  |  |
|  |  |  |  |  |
| 6.10 | Which nutritious items she should especially include in her diet?  MULTIPLE OPTIONS  (DO NOT READ OPTIONS AND PROBE)  कौन सी पौष्टिक/ताकतवाली चीज़ें खानी चाहिए? | Green vegetables  Fruits  Fish and meat(if she can)  Eggs  Milk  Pulses and beans  Other | 1  2  3  4  5  6  7 | |
| 6.11 | Do you think a pregnant woman should not eat certain food items?  क्या कुछ ऐसी भी चीज़ें हैं जो गर्भवती महिला को नहीं खानी चाहिए? | Yes  No  Can't say | 1  2 Go to 6.13  999 | |
|  |  |  |  |  |
|  |  |  |  |  |
| 6.12 | Which items she should not eat?  MULTIPLE OPTIONS  (DO NOT READ OPTIONS AND PROBE)  कौन सी चीज़ें नहीं खानी चाहिए? | Papaya  Sour food  Fried food  Spicy food  Other  Can't say | 1  2  3  4  5  999 | |
| 6.13 | Do you think a pregnant woman needs rest, and possibly take a nap in the afternoon?  क्या गर्भवती महिला को आराम की ज़रूरत होती है और हो सके तो उन्हें दोपहर में आराम करना चाहिए/सो जानाचाहिए ? | Yes  No  Can't say | 1  2  999 | |
| 6.14 | Do you think, little bleeding/ spotting during pregnancy require medical attention?  अगर गर्भावस्था के दौरान थोड़ा बहुत खून आये या धब्बे दिखें, तो क्या डॉक्टर को दिखाना चाहिए? | Yes  No  Can't say | 1  2  999 | |
|  |  |  |  |  |
|  |  |  |  |  |
| 6.15 | Do you think, swelling of hands and feet and face is alright during pregnancy and does not require medical attention?  अगर गर्भावस्था के दौरान हाथ, पैरों या चेहरे पे सूजन आये, तो क्या डॉक्टर को दिखाना चाहिए? | Yes  No  Can't say | 1  2  999 | |
| 6.16 | If there is a good *dai* available nearby, do you think then a woman can have delivery at home?  क्या आपको लगता कि अगर अच्छी दाई घर केआस पास है तो महिला घर पर प्रसव करवा सकती है ? | Yes  No  Can't say | 1  2  999 | |
| 6.17 | Are you aware of *Janani Suraksha Yojana*?  क्या आपको जननी सुरक्षा योजना के  बारे में पता है ? | Yes  No | 1  2 | |
| 6.18 | Are you aware of *Janani Shishu Suraksha Karyakram*?  क्या आपको जननी शिशु सुरक्षा कार्यक्रम के बारे में पता है? | Yes  No | 1  2 | |
| 6.19 | Have you enrolled for any of these?  क्याआपनेइनमेंसेकिसीभीयोजनामेंअपनानामलिखवायाहै? | Yes  No | 1  2 | |
| 6.20 | Do you know the signs of delivery time approaching?  आपकोकैसेपताचलेगाकिप्रसवकासमयआगयाहै? क्याआपउसकेलक्षणबतासकतीहैं? | Yes  No  DK | 1  2  999 | |
| 6.21 | Before you became pregnant this time, were you using any family planning method?  इस बार गर्भवती होने से पहले आपकिसी भी गर्भनिरोधक का इस्तमाल कर रही थीं? | Yes  No | 1  2 Go to 6.23 | |
| 6.22 | Which family planning method were you using?  (DO NOT READ OPTIONS AND PROBE)  आप कौन से गर्भनिरोधक का इस्तमाल कर रही थीं? | IUD/Loop/Copper T  Oral Pill  Condom  Injections (DMPA)  Traditional method  No response | 1  2  3  4  5  999 | |
| 6.23 | After you deliver this baby, would you like to adopt a family planning method?  इस बच्चे को जन्म देने के बाद, क्या आप गर्भनिरोधक का इस्तमाल करना चाहती हैं? | Yes  No  Can't say | 1  2 Go to 6.27  999 | |
| 6.24 | Which family planning method have you thought of adopting after this delivery?  (DO NOT READ OPTIONS AND PROBE)  इस बच्चे को जन्म देने के बाद आप कौनसे गर्भनिरोधक का इस्तमाल करना चाहती हैं? | Female Sterilization  Male Sterilization/NSV  IUD/Loop/Copper T  Oral Pill  Condom  Injections (DMPA)  Traditional method  Not decided | 1  2  3  4  5  6  7  999 | |
| 6.25 | Have you discussed with your husband about family planning method to be used after delivery?  क्या आपने इस बच्चे कोजन्म देने के बाद, गर्भनिरोधक इस्तमाल करने के बारे में अपने पति से बात की है? | Yes  No | 1  2 | |
| 6.26 | Who mainly motivated you to use family planning method after delivery?  बच्चे को जन्म देने के बाद, गर्भनिरोध कइस्तमाल करनेके बारे में आपको खासतौर पर किसने सलाह दी? | CHV  AWW  ANM  Doctor  Self  Family members  Friends/Neighbours/Relatives | 1  2  3  4  5  6  7 | |
| 6.27 | Do you know what should be the ideal gap between two children?  दो बच्चोंके बीच में कितना अंतर रखना सही होता है? | 3 years or more  Any other responses  DK | 1  2  999 | |
|  | ***7. Awareness of child health care*** | | | |
|  | There are many customs and practices regarding breastfeeding, complementary feeding, and child care that elderly women in the family or neighbors and others tell. We would like to know your opinion regarding these practices.  बच्चे की देखभाल के बहुत सारे रीती-रिवाज़ होते हैं, अब मैं आपसे उसके बारे में सवाल पूछूंगी\| | | | |
| 7.1 | Do you think , a newborn baby should be given some sweet item like honey, sugar water as the first item after birth.?  क्याआपको लगता है कि बच्चेके जन्म के बाद, उसको सबसे पहले कुछ मीठा जैसे शहद, चीनी का पानी इत्यादि देना चाहिए? | Yes  No  Can't say / not sure | 1  2  999 | |
|  |  |  |  |  |
|  |  |  |  |  |
| 7.2 | When should the mother start breastfeeding a newborn baby?  नवजात शिशुको माँ का दूध कब पिलाना शुरू करना चाहिए? | Immediately after birth/ Within one hour  Any other responses  DK | 1  2  999 | |
| 7.3 | If a newborn baby is not able to suckle properly, or suckles only for a short time, do you think, she should be then given outside milk?  अगर बच्चा माँ का दूध ठीक से नहीं पी पाये या थोड़े समय के लिए ही पिए, तो क्या उसे बाहर/ऊपर का दूध पिलाना शुरू कर देना चाहिए? | Yes  No  Can't say/not sure | 1  2  999 | |
| 7.4 | Do you think a new born baby should be given water?  क्या नवजात शिशु को पानी पिलाना चाहिए ? | Yes  No  Can't say | 1  2  999 | |
| 7.5 | At what age baby needs to be given something other than mother’s milk?  **किस उम्र में बच्चे को माँ के दूध के साथ खाना देना शुरू करना चाहिए?** | From 6 Months  Any other responses  Don't know | 1  2  999 | |
|  |  |  |  |  |
| 7.6 | Is it alright to breastfeed the baby even after the baby starts eating other food?  अगर बच्चा माँ के दूधके अलावा **खाना** खाने लगे, तो क्या उसे फिरभी माँ का दूध पिलाते रहना सही है? | Yes  No  Can't say | 1  2  999 | |
| 7.7 | How would you know that a baby requires medical help? Can you name a few symptoms?  आपको कैसे पता चलेगा की बच्चे को डॉक्टर को दिखाने की ज़रूरत है? क्या आप कुछ लक्षण बता सकती हैं? | Fever  Cough and cold  Cold body  Breathing difficulty  Jaundice  Baby not taking feed  Baby listless/not active  Diarrhea  Don't know | 1  2  3  4  4  5  6  7  999 | |
| 7.8 | What should be the weight of a normal baby at birth?  पैदा होने के समय एक सामान्य बच्चे का वज़न कितना होना चाहिए? | >= 2.5 kgs  Any other responses  DK | 1  2  999 | |
| 7.9 | Do you know a baby needs to be given some vaccines?  **क्या आपको पता है किबच्चे को कुछ टीके लगवाने की ज़रूरत पड़ती है?** | Yes  No  DK | 1  2 Go to 7.11  999 | |
| 7.10 | Do you know names of a few vaccines given to babies?  MULTIPLE OPTIONS  (DO NOT READ OPTIONS AND PROBE)  **क्या आपको बच्चो को दिए जाने वाले कुछ टीकों के नाम पता हैं?** | BCG  DPT  POLIO  measles  Other______________________  DK | 1  2  3  4  5  999 | |
| 7.11 | Do you know how many doses of different vaccinations are given to babies?  (READ ALL OPTIONS)  **क्या आपको पता है कि अलग अलग टीकों कीकितनी खुराक बच्चो को दी जाती हैं?** | BCG --------  DPT --------  Polio -------  Measles ------ | 1  2  3  4 | |
| 7.12 | If a baby misses one or two doses of any vaccine, do you think it will not harm the baby?  क्या आपको लगता है कि अग बच्चे को टीके की एक – दो खुराक नदी जाये तो उससे बच्चे को कोई नुकसान नहीं होगा? | Yes, no harm  No, it will harm  Can't say | 1  2  999 | |
| 7.13 | Do you think feeding a baby that has loose motions, aggravates diarrhea?  क्या दस्त के दौरान बच्चे को माँ का दूध पिलाने या खाना खिलाने से दस्त बढ़ जायेगा? | Yes  No  Can't say | 1  2  999 | |
| 7.14 | Do you think baby needs to be weighed periodically?  क्या समय समय पर बच्चे का वज़न लेते रहना चाहिए? | Yes  No  Can't say | 1  2  999 | |
| 7.15 | Do you think if neighbors/ friends get to know about your child’s weight, then child gets ill?  **अगर बच्चे के वज़न के बारे में पड़ोसी / दोस्तों को पता चलता है तो क्या आपको लगता है बच्चा बीमार हो जाता है ?** | Yes  No  Can't say | 1  2  999 | |
|  | ***8. Maternal Health Care Seeking Behavior*** | | | |
| 8.1 | Have you registered with any hospital/ health center/clinic for antenatal care services?  गर्भावस्था के दौरान जांच के लिए क्या आपने किसी अपस्पताल/ स्वास्थ्य सेवाकेंद्र/ क्लिनिक पर अपना नाम लिखवाया है/ पंजीकरण करवाया है? | Yes  No  Plan to register | 1  2. Go to 8.8  3. Go to 8.6 | |
| 8.2 | Where did you go for getting antenatal care?  MULTIPLE OPTIONS  (DO NOT READ OPTIONS AND PROBE)  गर्भावस्था के दौरान जांच के लिए आप कहाँ जाती हैं? | Govt/ Municipal hosp  Private hospital  Health post  Private doctor | 1  2  3  4 | |
| 8.3 | Did you get a card/papers (Mother & Child Protection card) at the time of registration? Can I see it?  नाम लिखवाने/ पंजीकरण के समय क्या आपको कोई कार्ड/पेपर्समिलेथे (माता एवं शिशु सुरक्षा कार्ड)? क्या मैं देख सकती हूँ ? | Yes  No  Yes, but could not show | 1  2  3 | |
| 8.4 | How many months pregnant were you when you first registered for ANC?  आप कितने महीने गर्भवती थीं जब आपने जांच के लिए अपना नाम लिखवाया /पंजीकरण करवाया था? | ______________Months  Do not remember | 999 | |
| 8.5 | The first time that you visited the doctor during your current pregnancy, did you go because you were pregnant or did you go because you had health problems?  आप इस गर्भावस्था में पहलीबार जब डॉक्टर के पास गयीं, तब आप इसलिए गयीं थीं कि आप गर्भवती थीं या आपको कोई स्वास्थ्य सम्बंधित परेशानी थी? | Went, because I was pregnant  Went, because of health problems  Both | 1  2  3 | |
| 8.6 | Who mainly advised/motivated you to get registered?  गर्भावस्था के दौरान अस्पताल में नाम लिखवाने/ पंजीकरण करवाने के लिए आपको खासतौर पर किसने सलाह दी? | CHV  AWW  ANM  Doctor  Self  Family members  Friends/Neighbours/Relatives  Not reported | 1  2  3  4  5  6  7  999 | |
| 8.7 | How many times did you go for antenatal check-ups so far, during this pregnancy?  इस गर्भावस्था के दौरान आप अभी तक कितनी बार जाँच के लिए गयीं ? | Number of ANC visits _________________  Not reported | 999 | |
| 8.8 | Did you receive any TT (Tetanus Toxoid) injection during this pregnancy?  क्या आपको इस गर्भावस्था के दौरान टी टी (टेटनस टॉक्साइड) का इंजेक्शन (सुई) लगा था ? | Yes  No  Don't remember | 1  2 Go to 8.11  999 | |
| 8.9 | *How many times did you receive TT injections?*  आपको कितनी बार टी टी की सुई लगी है ? | Number ______  Don't remember | 999 | |
| 8.10 | Who mainly motivated you to take TT injection?  टी टी की सुई लेने के लिए आपको खासतौरपर किसने सलाह दी? | CHV  AWW  ANM  Doctor  Self  Family members  Friends/Neighbours/Relatives | 1  2  3  4  5  6  7 | |
| 8.11 | Did you take Calcium tablets during the pregnancy?  क्या आपने गर्भावस्था के दौरानकैल्शियम की गोलियां ली है? | Yes  No  Don't remember | 1  2 Go to 8.13  999 | |
| 8.12 | Who mainly motivated you to take calcium supplementation?  कैल्शियम की गोलियां लेने के लिए आपको खासतौरप रकिसने सलाह दी? | CHV  AWW  ANM  Doctor  Self  Family members  Friends/Neighbours/Relatives | 1  2  3  4  5  6  7 | |
| 8.13 | Did you receive or purchase any iron folic acid (IFA) tablets/syrup bottles during the pregnancy?  गर्भावस्थाके दौरान क्या आपको आइरन फोलिकएसिड गोलियां/ बोतल मिली या आपनेखरीदी? | Yes  No  Don't remember | 1  2 Go to 8.18  3 | |
| 8.14 | If yes, how many tablets/syrup bottles of IFA did you receive /purchase so far?  अभीतक आपको आइरन फोलिक एसिडकी कितनी गोलियां/बोतल मिली हैं/खरीदीहैं? | Tablets  Bottles  Don't remember | 999 | |
| 8.15 | How many tablets/bottles of IFA did you consume, so far?  आपने अभी तक आइरन फोलिक एसिड की कितनी गोलियां खायीं / बोतल पी ली हैं? | Tablets  Bottles  Don't remember | 999 | |
| 8.16 | In which month of pregnancy did you start taking IFA tablets/ syrup?  गर्भावस्था के कौन से महीने में आपनेआइरन फोलिक एसिड की गोलियां/ सिरप लेना शुरू किया था ? | ______ month  Don't remember | 999 | |
| 8.17 | Who mainly motivated you to take IFA tablets/ syrup?  आइरन फोलिक एसिड की गोलियां/ सिरप लेनेके लिए आपको खासतौरपर किसने सलाह दी? | CHV  AWW  ANM  Doctor  Self  Family members  Friends/Neighbours/Relatives | 1  2  3  4  5  6  7 | |
| 8.18 | As compared to your usual intake, are you eating more / less/usual quantity of food during your pregnancy?  गर्भावस्थामें क्या आप पहलेसे ज़्यादा खाना खा रही हैं, कम खाना खा रही हैं या जितना खाती थी उतनाही खाना चाहिए? | More amount  Same amount  Less amount  Can't say | 1  2  3  999 | |
| 8.19 | Which nutritious items are you especially including in your diet during this pregnancy?  MULTIPLE OPTIONS  (DO NOT READ OPTIONS AND PROBE)  कौनसी पौष्टिक/ताकतवाली चीज़ें आप खासतौरपर खा रही हैं? | Green vegetables  Fruits  Fish and meat  Eggs  Milk  Pulses and beans  Other  Can't say | 1  2  3  4  5  6  7  999 | |
| 8.20 | Who mainly motivated/ suggested you to include these items in your diet?  पौष्टिक/ताकतवाली चीज़ें खानेकी सलाह आपको खासतौर पर किसने दी? | CHV  AWW  ANM  Doctor  Self  Family members  Friends/Neighbours/Relatives | 1  2  3  4  5  6  7 | |
| 8.21 | During pregnancy, are you resting more/taking afternoon nap regularly?  गर्भावस्था के दौरान क्या आप ज्यादा आराम करती हैं/ आपने दोपहर में सोना शुरूकियाहै? | Yes, regularly  Yes, sometimes  No  Can't say | 1  2  3 Go to 8.23  999 | |
| 8.22 | Who mainly motivated/ suggested you to take rest/ afternoon nap?  ज्यादा आराम/दोपहर में सोने की सलाह आपको खासतौरपर किसने दी? | CHV  AWW  ANM  Doctor  Self  Family members  Friends/Neighbours/Relatives | 1  2  3  4  5  6  7 | |
| 8.23 | Did your husband accompany you during any of your ante-natal check-up?  क्या आपके पतिगर्भावस्थाके दौरान जाँचकेलिए आपके साथ जाते हैं? | Yes, regularly  Yes, mostly  Yes, sometimes  No | 1  2  3  4 | |
| 8.24 | During pregnancy did you have swelling on hands/ feet/ face?  क्या आपको गर्भावस्था के दौरान हाथ, पाव एवं चेहरे पर सूजन आई? | Yes  No | 1  2 Go to 8.27 | |
| 8.25 | Did you consult doctor for this?  क्याइसकेलिएआपनेडॉक्टरसेसलाहली? | Yes  No | 1  2 Go to 8.27 | |
| 8.26 | Where did you go for consultation?  आप डॉक्टरकी सलाह के लिए कहाँ गए थे? | Gov. / Municipal hosp  Private hospital  Health post  Private clinic  No response | 1  2  3  4  999 | |
| 8.27 | During pregnancy do you feel excessively tired?  गर्भावस्थाके दौरान क्या आपको थकान ज़्यादा महसूस होती है? | Yes  No  Can't say | 1  2  999Go to 8.30 | |
| 8.28 | Did you consult doctor for this?  क्या इसके लिए आपने डॉक्टरसे सलाह ली? | Yes  No | 1  2 Go to 8.30 | |
| 8.29 | Where did you go for consultation?  आप डॉक्टरकी सलाह के लिए कहाँ गए थे? | Gov. / Municipal hosp  Private hospital  Health post  Private clinic  No response | 1  2  3  4  999 | |
| 8.30 | During pregnancy did you have spotting/ bleeding?  गर्भावस्थाके दौरान क्या आपको कभी खून आया/धब्बे दिखे? | Yes  No  Don't remember | 1  2 Go to 8.33  999 | |
| 8.31 | Did you consult doctor for this?  क्या इसके लिए आपने डॉक्टरसे सलाह ली? | Yes  No | 1  2 Go to 8.33 | |
| 8.32 | Where did you go for consultation?  आप डॉक्टरकी सलाह के लिए कहाँ गए थे? | Gov. / Municipal hosp  Private hospital  Health post  Private clinic  No response | 1  2  3  4  999 | |
| 8.33 | During pregnancy did you often have severe headache?  गर्भावस्थाके दौरान क्या आपको सरमें बहुत तेज़ दर्द होता है? | Yes  No  Can't say | 1  2 Go to 8.36  999 | |
| 8.34 | Did you consult doctor for this?  क्याइसकेलिएआपनेडॉक्टरसेसलाहली? | Yes  No | 1  2 Go to 8.36 | |
| 8.35 | Where did you go for consultation?  आप डॉक्टरकी सलाह के लिए कहाँ गए थे? | Gov. / Municipal hosp  Private hospital  Health post  Private clinic  No response | 1  2  3  4  999 | |
| 8.36 | Have you / did you plan to deliver in hospital?  क्याआपअस्पतालमें प्रसव करानेका सोच रही हैं? | Yes  No  Not decided | 1  2 Go to 8.38  999 | |
| 8.37 | Who mainly motivated you to plan delivery in the hospital?  अस्पतालमें प्रसव कराने की सलाह आपको खासतौरपर किसने दी? | CHV  AWW  ANM  Doctor  Self  Family members  Friends/Neighbours/Relatives | 1  2  3  4  5  6  7 | |
| 8.38 | Have you made arrangements for saving money for delivery and other expenses?  क्या आपने डिलीवरी और अन्य ख़र्चों के लिए पैसे की बचत या इंतज़ाम किया है? | Yes  No  Plan to do so | 1  2 go to 8.40  3 | |
| 8.39 | Who mainly motivated you to plan for money for delivery?  पैसे की बचत या इंतज़ाम करने की सलाह आपको खासतौरपर किसने दी? | CHV  AWW  ANM  Doctor  Self  Family members  Friends/Neighbours/Relatives | 1  2  3  4  5  6  7 | |
| 8.40 | Have you made arrangements for a vehicle to reach hospital in case of any emergency?  क्याआपने अचानक ज़रूरत पड़ने पर, अस्पताल जानेके लिए किसी वाहन/साधनका इंतज़ाम किया है? | Yes  No  Plan to do so | 1  2  3 | |
| 8.41 | Who mainly motivated you to plan other arrangements for delivery?  बाकी सब इंतेज़ाम करने की सलाह आपको खासतौरपर किसने दी? | CHV  AWW  ANM  Doctor  Self  Family members  Friends/Neighbors/Relatives | 1  2  3  4  5  6  7 | |
| 1.18_A | Status of the Interview | Complete  Incomplete | 1  2 | |
| 1.19 | Enrolled for mMitra | Yes  No | 1  2 | |
| 1.20 | Respondent Category  (DO NOT ASK, PLEASE FILL IT ON YOUR OWN) | Intervention group  Control group | 1  2 | |
| 1.21 | Preferred language for voice call | English  Marathi  Hindi | 1  2  3 | |
| 1.22 | Preferred timing to receive voice call | 9-11 am  11-1 am  1-3 pm  3-5 pm  5-7 pm  7-9 pm | 1  2  3  4  5  6 | |
