# Supplementary material for: Effects of an mHealth voice message service (mMitra) on maternal health knowledge and practices of low-income women in India: findings from a pseudo-randomized controlled trial
Source: BMC Public Health. 2020 Jun 1;20:820. doi: 10.1186/s12889-020-08965-2 (PMC7268375; doi:10.1186/s12889-020-08965-2)
Supplement: Supplementary file 2 — Additional file 2 Supplement 2. Time 2 (Post-Partum) Survey. Questionnaire SECOND INTERVIEW: Impact Evaluation of mMitra. [file 12889_2020_8965_MOESM2_ESM.docx]

**Supplement 2. Time 2 (Post-Partum) Survey**

*For Research Purposes Only*

**Questionnaire SECOND INTERVIEW: Impact Evaluation of mMitra**

| **Sr. No.** | **Questions** | **Response** | **Code** |
| --- | --- | --- | --- |
|  | ***1. Identification Details*** |  |  |
|  | ***Investigator code*** |  |  |
| 1.1a | Date of the Interview  **(DO NOT ASK, PLEASE FILL IT ON YOUR OWN)** | dd/mm/yyyy |  |
| 1.2 | Serial number of the Respondent  **(DO NOT ASK, TO BE COPIED FROM FIRST INTERVIEW SCHEDULE)**  **उत्तर** |  |  |
|  | Unique Id from First interview |  |  |
| 1.3 | Name of the Respondent  भुराखतदायाचेनाल  **(DO NOT ASK, TO BE COPIED FROM FIRST INTERVIEW SCHEDULE)** | _________________ |  |
|  | Enrolled for mMitra  **(DO NOT ASK, TO BE COPIED FROM FIRST INTERVIEW SCHEDULE)** | Yes  No | 1  2 |
| 1.1 | Date of enrollment in mMitra  **(DATE OF FIRST INTERVIEW. DO NOT ASK, TO BE COPIED FROM FIRST INTERVIEW SCHEDULE)** | dd/mm/yyyy |  |
| 1.10 | Name of the Interviewer | ______________________________ |  |
|  | *Congratulations. I am now going to ask you question about your new baby and your family* | | |
| 1.16 | Date of delivery | dd/mm/yyyy |  |
| 1.17 | Outcome of delivery | Live birth  Still birth  Miscarriage  Born alive but died later  Mother died | 1  2  3  4  5 STOP |
| 1.18 | Sex of the baby (if alive) | Male  Female | 1  2 |

| 3 | **3. Individual Information** | ***Now, I am going to ask you some questions about yourself and your husband*** |  | |
| --- | --- | --- | --- | --- |
| 3.9 | How often do you watch television? | Almost every day  Once a week  Occasionally/Rarely  Not at all | 1  2  3  4 | |
| 5.0 | *Now, I am going to ask you some questions about relationship you share with your husband and other household members.* | |  | |
| 5.2 | Does your husband smoke cigarettes or bidis? | Yes  No | 1  2 | |
| 5.3 | Does he smoke at home or outside? | Home  Outside | 1  2 | |
| 5.12 | Do you share your baby’s health problems, even the smaller ones, with your husband? | Yes  No |  | |
| 5.13 | When your baby is unwell, does your husband go with you to doctor? | Yes, most of the times  Yes, if it is serious  No, because he is busy  Not at all  No need |  | |
| 5.14 | Do you share your baby’s health problems, with other household members? | Yes  No |  | |
| 6.0 | ***Awareness of Maternal Health Care*** |  |  | |
|  | There are many customs and practices regarding diet, rest, health care to be followed during pregnancy. Now I am going to ask you some questions regarding your beliefs about these practices. | |  | |
| 6.1 | Pregnancy and child birth is a natural phenomenon. Do you think any medical help is required? | Yes  No  Can't say | 1  2  999 | |
| 6.2 | If a woman does not have any health problem during pregnancy, do you think then also she should see a doctor? | Yes  No  Can't say | 1  2  999 | |
|  |  |  |  |  |
|  |  |  |  |  |
| 6.3 | During which month of pregnancy should a woman first see a doctor? | First Trimester  Any other responses  Can't say/DK | 1  2  999 | |
| 6.6 | Do you know which tablets women have to take for supplementation? | Iron  Calcium  Other  DK | 1  2  3  999 | |
| 6.8 | As compared to her usual intake, do you think a pregnant woman should eat more / less/usual quantity of food? | More food  Less food/ Same as usual  Can't say | 1  2  999 | |
| 6.10 | Which nutritious items she should especially include in her diet?  MULTIPLE OPTIONS  (DO NOT READ OPTIONS AND PROBE) | Green vegetables  Fruits  Fish and meat(if she can)  Eggs  Milk  Pulses and beans  Other | 1  2  3  4  5  6  7 | |
| 6.12 | Which items she should not eat?  MULTIPLE OPTIONS  (DO NOT READ OPTIONS AND PROBE) | Papaya  Sour food  Fried food  Spicy food  Other  Can't say | 1  2  3  4  5  999 | |
| 6.13 | Do you think a pregnant woman needs rest, and possibly take a nap in the afternoon? | Yes  No  Can't say | 1  2  999 | |
| 6.14 | Do you think, little bleeding/ spotting during pregnancy require medical attention? | Yes  No  Can't say | 1  2  999 | |
| 6.19 | Did you enroll for JSY ? | Yes  No | 1  2 | |
| 6.23 | After you deliver this baby did you adopt family planning / are you planning to adopt family planning? | Yes, adopted  Not yes adopted but planning to adopt soon  Not planning  Not decided | 1  2  3 Go to 6.27  999 | |
| 6.24 | Which family planning method have you adopted / thought of adopting? | Female Sterilization  ICUD  Oral Pill  Condom  Injections (DMPA)  Traditional method  Not decided | 1  2  3  4  5  6  999 | |
| 6.25 | Did/ Have you discussed with your husband about family planning method you adopted / thought of adopting? | Yes  No | 1  2 | |
| 6.26 | Who mainly motivated you to use family planning method after delivery? | CHV  AWW  ANM  Doctor  Self  Family members  Friends/Neighbours/Relatives  mMitra | 1  2  3  4  5  6  7  8 | |
| 6.27 | Do you know what should be the ideal gap between two children? | 3 years or more  Any other responses  DK | 1  2  999 | |
|  | ***7. Awareness of child health care*** |  |  | |
|  | There are many customs and practices regarding breastfeeding, complementary feeding, and child care that elderly women in the family or neighbors and others tell. We would like to know your opinion regarding these practices | |  | |
| 7.1 | Do you think that the first item after birth, a newborn baby should be given some sweet item like honey, sugar water etc.? | Yes  No  Can't say / not sure | 1  2  999 | |
|  |  |  |  |  |
|  |  |  |  |  |
| 7.2 | When should the mother start breast  feeding a newborn baby? | Immediately after birth/ Within one hour  Any other responses  DK | 1  2  999 | |
| 7.3 | If a newborn baby is not able to suckle properly, or suckles only for a short time, do you think, and then she should be given outside milk? | Yes  No  Can't say/not sure | 1  2  999 | |
| 7.4 | Do you think a new born baby should be given water? | Yes  No  Can't say | 1  2  999 | |
| 7.5 | At what age baby needs to be given something other than mother’s milk? | From 6 Months  Any other responses  Don't know | 1  2  999 | |
|  |  |  |  |  |
| 7.6 | Is it alright to breastfeed the baby even after the baby starts eating other food? | Yes  No  Can't say | 1  2  999 | |
| 7.7 | How would you know that a baby requires medical help? Can you name a few symptoms?    MULTIPLE OPTIONS  (DO NOT READ OPTIONS AND PROBE) | Fever  Cough and cold  Cold body  Breathing difficulty  Jaundice  Baby not taking feed  Baby listless/not active  Diarrhea  Don't know | 1  2  3  4  5  6  7  8  999 | |
| 7.8 | What should be the weight of a normal baby at birth? | >= 2.5 kgs  Any other responses  DK | 1  2  999 | |
| 7.17 | Do you know how much your baby weighed at birth | _______Kg  Not weighed  Don't know | 8  999 | |
| 7.9 | Do you know, a baby needs to be given some vaccines? | Yes  No  DK | 1  2 go to 7.11  999 | |
| 7.10 | Do you know names of a few vaccines given to babies? | BCG  DPT  POLIO  measles  Other______________________  DK | 1  2  3  4  5  999 | |
| 7.11 | Do you know how many doses of different vaccinations are given to babies?  (Read All options) | BCG --------  DPT --------  Polio -------  Measles ------  DK | 999 | |
| 7.12 | If a baby misses one or two doses of any vaccine, do you think it will not harm the baby? | Yes, No harm  No, it will harm  Can't say | 1  2  999 | |
| 7.13 | Do you think feeding a child that has loose motions, aggravates diarrhea? | Yes  No  Can't say | 1  2  999 | |
| 7.14 | Do you think, baby needs to be weighed periodically? | Yes  No  Can't say | 1  2  999 | |
| 7.15 | Do you think if neighbors/ friends get to know about your baby's weight, then baby gets ill? | Yes  No  Can't say | 1  2  999 | |
|  | ***8. Maternal Health Care Seeking Behavior*** | |  | |
| 8.2 | Where all did you go for getting antenatal care?  MULTIPLE OPTIONS  (DO NOT READ OPTIONS AND PROBE) | Govt/ Municipal hosp  Private hospital  Health post  Private doctor  nowhere | 1  2  3  4  5 | |
| 8.4 | How many months pregnant were you when you first registered for ANC? | ______________Months |  | |
| 8.6 | Who mainly advised/motivated you to get registered? | CHV  AWW  ANM  Doctor  Self  Family members  Friends/Neighbours/Relatives  mMitra | 1  2  3  4  5  6  7  8 | |
| 8.7 | How many times did you go for antenatal check-ups so far, during this pregnancy ? | Number of visits __  Don't remember | 999 | |
| 8.8 | Did you receive any TT (Tetanus Toxoid) injection during pregnancy? | Yes  No  Don't remember | 1  2 go to 8.11  999 | |
| 8.9 | How many times did you receive TT? | Number -----  Don't remember | 999 | |
| 8.11 | Did you take Calcium tablets during pregnancy? | Yes  No  Don't remember | 1  2 go to 8.13  999 | |
| 8.12 | Who mainly motivated / told you to take calcium supplementation? | CHV  AWW  ANM  Doctor  Self  Family members  Friends/Neighbours/Relatives  mMitra | 1  2  3  4  5  6  7  8 | |
| 8.13 | Did you receive or purchase any iron folic acid (IFA) tablets/bottles during the pregnancy? | Yes  No  Don't remember | 1  2 go to 8.18  999 | |
| 8.14 | If yes, how many tablets/bottles of IFA did you receive /purchase? | ________________  Don't remember | 999 | |
| 8.15 | How many tablets/bottles of IFA did you consume during pregnancy? | _______  Don't remember? | 999 | |
| 8.16 | In which month of pregnancy did you start taking IFA tablets/ syrup? | ______ month  Don't remember | 999 | |
| 8.17 | Who mainly motivated you to take IFA tablets/ syrup? | CHV  AWW  ANM  Doctor  Self  Family members  Friends/Neighbours/Relatives  mMitra | 1  2  3  4  5  6  7  8 | |
| 8.18 | As compared to your usual intake, did you eat more / less/usual quantity of food during your pregnancy? | More amount  Same amount  Less amount  Can't say | 1  2  3  999 | |
| 8.19 | Which nutritious items did you especially include in your diet? | Green vegetables  Fruits  Fish and meat  Eggs  Milk  Pulses and beans  Other  Can't say | 1  2  3  4  5  6  7  999 go to 8.21 | |
| 8.20 | Who mainly motivated/ suggested you to include these items in your diet? | CHV  AWW  ANM  Doctor  Self  Family members  Friends/Neighbours/Relatives  mMitra | 1  2  3  4  5  6  7  8 | |
| 8.21 | During pregnancy, did you rest more/taking afternoon nap regularly? | Yes, regularly  Yes, sometimes  No  Can't say | 1  2  3 Go to 8.23  999 | |
| 8.22 | Who mainly suggested that you take rest/afternoon nap? | CHV  AWW  ANM  Doctor  Self  Family members  Friends/Neighbours/Relatives  mMitra | 1  2  3  4  5  6  7  8 | |
| 8.23 | Did your husband accompany you during any of your ante-natal check-up? | Yes, regularly  Yes, mostly  Yes, sometimes  No | 1  2  3  4 | |
| 8.24 | During pregnancy did you have swelling on hands/ feet/ face? | Yes  No | 1  2 Go to 8.27 | |
| 8.25 | Did you consult doctor for this? | Yes  No | 1  2 Go to 8.27 | |
| 8.26 | Where did you go for consultation? | Gov. / Municipal hosp  Private hospital  Health post  Private clinic  No response | 1  2  3  4  999 | |
| 8.27 | During pregnancy were you feeling excessively tired? | Yes  No  Can't say | 1  2 Go to 8.30  999 | |
| 8.28 | Did you consult doctor for this? | Yes  No | 1  2 Go to 8.30 | |
| 8.29 | Where did you go for consultation? | Gov. / Municipal hosp  Private hospital  Health post  Private clinic  No response | 1  2  3  4  999 | |
| 8.30 | During pregnancy did you have spotting/ bleeding? | Yes  No  Don't remember | 1  2 Go to 8.33  999 | |
| 8.31 | Did you consult doctor for this? | Yes  No | 1  2 Go to 8.33 | |
| 8.32 | Where did you go for consultation | Gov. / Municipal hosp  Private hospital  Health post  Private clinic  No response | 1  2  3  4  999 | |
| 8.33 | During pregnancy did you often have severe headache? | Yes  No  Can't say | 1  2 Go to 8.36  999 | |
| 8.34 | Did you consult doctor for this? | Yes  No | 1  2 Go to 8.36 | |
| 8.35 | Where did you go for consultation? | Gov. / Municipal hosp  Private hospital  Health post  Private clinic  No response | 1  2  3  4  999 | |
| 8.36 | Did you plan to deliver in hospital? | Yes  No  Not decided | 1  2 Go to 8.38  999 | |
| 8.37 | Who mainly motivated to plan delivery in the hospital? | CHV  AWW  ANM  Doctor  Self  Family members  Friends/Neighbours/Relatives  mMitra | 1  2  3  4  5  6  7  8 | |
| 8.38 | Did you make arrangements for saving money for delivery and other expenses? | Yes  No  Plan to do so | 1  2 Go to 8.40  3 | |
| 8.39 | Who mainly motivated you to plan for money for delivery? | CHV  AWW  ANM  Doctor  Self  Family members  Friends/Neighbours/Relatives  mMitra | 1  2  3  4  5  6  7  8 | |
| 8.40 | Did you make arrangements for a vehicle to reach hospital in case of any emergency? | Yes  No  Plan to do so | 1  2  3 | |
| 8.41 | Who mainly motivated you to plan other arrangements for delivery? | CHV  AWW  ANM  Doctor  Self  Family members  Friends/Neighbours/Relatives  mMitra | 1  2  3  4  5  6  7  8 | |
| 9.0 | **Delivery care seeking behaviour and initiation of breast feeding** | | | |
| 9.1 | For how many hours you were in labor? | _________hours  DK | 999 | |
| 9.2 | Where did you deliver your baby | Gov / Municipal hospital  Private hospital  Home | 1 Go to 9.8  2  3 | |
| 9.3 | Why did you not go to hospital for the delivery? | No time to reach hospital  No money  Family opposed  Hospital Facility not good  Previous delivery at home  No need was felt  Can't say | 1  2  3  4  5  6  999 | |
| 9.4 | Who conducted your delivery areastt home? | Doctor (GOVT/PVT)  ANM/MIDWIFE/LHV/NURSE  Birth Attendant(Dai)  Family member  Myself | 1  2  3  4  5 | |
| 9.5 | How did you cut the umbilical cord? | New blade  Kitchen knife  Other instrument  Don't know | 1  2  3  999 | |
| 9.6 | Was anything applied on the umbilical cord stump? | Yes  No  DK | 1  2  999 | |
| 9.7 | Did you go to hospital/ consult doctor within two days of delivery? | Yes  No | 1  2 | |
| 9.8 | If hospital delivery: How long did you wait to go to hospital after labor pain started? | ______ hours  Immediately  Don't remember | 0  999 | |
| 9.9 | How did you reach the hospital? | Walking  Public transport/taxi  Private vehicle  Ambulance  Don't remember | 1  2  3  4  999 | |
| 9.10 | Was it a normal delivery or caesarian section? | Normal delivery  Caesarian section | 1  2 | |
| 9.11 | How long (in hours) did you stay in hospital? | Less than 24 hours  24-36 hours  37-48 hours  48-72 hours  More than 72 hours  Don't remember | 1  2  3  4  5  999 | |
| 9.12 | Did you experience any of the following symptoms within a week of delivery?  MULTIPLE OPTIONS  (READ OPTIONS AND PROBE) | Excessive bleeding with blood clots  Foul smelling bleeding  High fever  Foul smelling discharge  Feeling faint/ dizzy  None | 1  2  3  4  5  999 GO TO 9.17 | |
| 9.13 | Where were you when you experienced these symptoms? | In Hospital  At Home | 1  2 | |
| 9.14 | Did you know that these are serious problems and needs visit to doctor? | Yes  No | 1  2 | |
| 9.15 | Did you consult a doctor or go to hospital for these symptoms? | Yes  No | 1  2 | |
| 9.16 | Who mainly advised you to consult doctor? | CHV  AWW  ANM  Doctor  Self  Family members  Friends/Neighbours/Relatives  mMitra | 1  2  3  4  5  6  7  8 | |
| 9.17 | Did you ever breastfeed the baby? | Yes  No | 1  2 GO TO 9.28 | |
| 9.18 | How long after birth did you first put the baby to the breast? | Immediately, with an hour  The same day  After two days  Can't say | 1 GO to 9.20  2  3  999 | |
| 9.19 | What was the reason you could not breastfeed the baby immediately? | Mother ill/Cesarean  Baby ill  Baby not able to suckle properly/ suckled only for a short time  No milk  Not aware  Can't say | 1  2  3  4  5  999 | |
| 9.20 | Who mainly motivated/ suggested you to breast feed the baby immediately? | CHV  AWW  ANM  Doctor  Self  Family members  Friends/Neighbours/Relatives  mMitra | 1  2  3  4  5  6  7  8 | |
| 9.21 | Did you feed the first yellow thick breast milk (Colostrum)/*Khees* to the baby? | Yes  No | 1  2 Go to 9.23 | |
| 9.22 | Who mainly motivated/ suggested to feed the first yellow thick breast milk (Colostrum)/*Khees* to the baby? | CHV  AWW  ANM  Doctor  Self  Family members  Friends/Neighbours/Relatives  mMitra | 1  2  3  4  5  6  7  8 | |
| 9.23 | In the first 3 days after delivery, was the baby given anything to drink other than breast milk? | Yes  No  DK | 1  2 Go to 9.25  999 Go to 9.26 | |
| 9.24 | What was given to the baby to drink?  MULTIPLE OPTIONS  (DO NOT READ OPTIONS AND PROBE) | Milk (other than breast milk)  Plain water  Formula/powder milk  Sugar water  Gripe water  Fruit juice  Honey  Ghutti  Other | 1  2  3  4  5  6  7  8  999 | |
| 9.25 | Who mainly motivated/ suggested you to not give the baby anything to drink other than breast milk? | CHV  AWW  ANM  Doctor  Self  Family members  Friends/Neighbours/Relatives  mMitra | 1  2  3  4  5  6  7  8 | |
| 9.26 | Was (NAME) breastfed yesterday during the day or at night? | Yes  No | 1  2 | |
| 9.27 | How many times yesterday during day or night, did the baby consume liquid other than breast milk? | ____  Cannot say | 999 | |
| 9.28 | Was the baby's health checked up after you and baby were discharged from the hospital, even if he/she was not ill? | Yes  No  Don't remember | 1  2  999 | |
| 9.29 | Did you get an advice on need for regular medical check-up of the baby? | Yes  No  Don't remember | 1  2 Go to 10  999 | |
| 9.30 | Who mainly advised you on need to get regular medical check-up of a young child? | CHV  AWW  ANM  Doctor  Self  Family members  Friends/Neighbours/Relatives  mMitra | 1  2  3  4  5  6  7  8 | |
|  | Respondent Category  **(DO NOT ASK, PLEASE FILL IT ON YOUR OWN)** | Intervention group  Control group | 1  2 GO TO HEALTH CARD | |
| 10. | mMitra Voice Call Service | Please ask Section 10 only to those who are enrolled in mMitra voice call service and therefore part of intervention group |  | |
| 10.1 | How many days after you were registered in mMitra voice call service did you start receiving mMitra voice calls? | Within 1 week  2 weeks  3-4 weeks  More than 4 weeks  Not received any call  Don't remember | 1  2  3  4  5 GO TO HEALTH CARD  999 | |
| 10.4 | Did you or any of your family members give a miss call to mMitra voice call service to inform them about your delivery? | Yes  No  DK | 1  2  999 | |
| 10.6 | Did you receive any call from mMitra voice call service to enquire about your date of delivery? | Yes  No  DK | 1  2  999 | |
| 10.7 | How many calls did you receive in the first week after your delivery? | Once every day  Once a week  Twice a week  Others  Don't remember | 1  2  3  4  999 | |
| 10.9 | How many calls did you listen to in last week? | 1 call  2 calls  3-6 calls  7 calls  Don't remember | 1  2  3  4  999 | |
| 10.10 | Did you listen to the voice calls yourself, or someone else heard it for you and then told you about the call? | Self  Husband  Mother in law  Others | 1  2  3  4 | |
| 10.11 | Did you listen to the voice calls alone, or with other family members? (probe) | Alone  With Husband  With Mother in law  Others | 1  2  3  4 | |
| 10.12 | Could you please tell us if you heard information about the following topics from mMitra voice calls during pregnancy? | Nutrition  Development of foetus  Anemia  Rest during pregnancy  Iron folic acid supplementation  Blood test  Calcium  Ante natal check up reminders  Sonography  HIV testing  Danger Signs during pregnancy  Identifying labour pains  Birth Preparedness  Cutting cord and dressing the stump  Family Planning  Sanitation and Hygiene  Breast Feeding  Colostrums  Sex determination  Others | Spontaneous  1  2  3  4  5  6  7  8  9  10  11  12  13  14  15  16  17  18  19  20 | Prompted  1  2  3  4  5  6  7  8  9  10  11  12  13  14  15  16  17  18  19  20 |

| 10.15 | How often do you listen to the full call? | Always  Often  Sometimes  Never  Can't say | 1  2  3  4  999 |
| --- | --- | --- | --- |
| 10.17 | If you miss mMitra voice call, what can you do to listen to the message? | Give a miss call  Give a miss call on "38"  Don’t know | 1  2  999 |
| 10.17.1 | Have you ever given missed call to listen to the message that you had missed? | Yes  No  Don’t Know | 1  2  999 |
| 10.27 | Did you find the calls useful? | Yes  No  Can't say | 1  2  999 |
| 10.29 Now I am going to ask you a few questions related to mMitra voice call service, please tell me how satisfied you are. | | | |
| 10.32a | *How satisfied are you with mMitra service?* | Very satisfied,  satisfied,  somewhat satisfied,  dissatisfied  very dissatisfied  can't say | 1  2  3  4  5  6 |
| **11. Information about Mobile Phone usage** | | | |
| 11.1 | Is the phone on which you receive mMitra voice calls functional currently? | Yes  No  DK/NR | 1  2  999 |
| 11.2 | On whose phone do you receive mMitra voice calls? | Self  Husband  Other Family members | 1  2  999 |
| 11.6 | What was the amount that you spent last time for recharging the phone? | ___________________ Rs  Don't remember  Don't know | 88  999 |
| 1.18_A | Status of the Interview | Complete  Incomplete | 1  2 |
